# The compressed workweek in public health and social care – is it sustainable?

**DOI:** 10.5271/sjweh.4276

**Published:** 2026-03-01

**Authors:** Dagfinn Matre

Is it a viable strategy to compress the workweek of public health and social care workers into fewer, longer shifts in exchange for fewer workdays? The consequences for both the quality of work and the employee seem to be unclear.

Sustaining public health and social care services to protect public welfare requires a stable workforce. Vulnerable patients benefit from continuity of care, trust, and emotionally secure caregiver interactions. A stable workforce also brings advantages to employers, boosting productivity and reducing costs related to sickness absence and employee turnover (1). However, the growing demand for care of the elderly in OECD countries clashes with a shrinking health and care workforce, driven by factors such as early retirements by women, disability, and a shortage of newly trained professionals (2, 3). This scenario creates a self-reinforcing cycle, where fewer staff face rising burdens, leading to retention challenges.

In Norway, the municipal health and care sector faces a doctor-certified sickness absence rate of 26% (around one third is reported to be work-related), notably higher than the national average of 7% (4). The majority (60%) of long-term sickness absence is due to musculoskeletal and mental diagnoses (4). The sector is also characterized by a higher proportion of workers leaving the active workforce due to health-related diagnoses than the national average (98 versus 66 per 10 000 workers) (4). Also here, musculoskeletal and mental diagnoses dominate. Prevention strategies targeting sickness absence, early retirement, and disability pension are needed to address the workforce shortage. Turnover intention is affected by family and social responsibilities but is also affected by organizational aspects, such as workload and working time arrangements, which are modifiable (5–7).

The health and care sector operates continuously, necessitating shift work, including irregular working hours. In Norway, around 90% of employees in this sector work outside regular daytime hours (4). One type of shift work is the compressed workweek (CWW), where employees work extended daily work hours compensated by a reduced number of workdays, without increasing the total weekly work hours. In a CWW, the worker extends his or her workdays either only during the weekend or throughout the workweek (8). In offshore drilling or industrial work, for example, 12-hour shifts are common. In the USA and the UK, the healthcare sector has adopted 12-hour shifts or even longer ones as part of a 2-shift system (9). In Norwegian municipal health and care services, CWW with extended daily hours has gained attention as a potential solution to workforce stability challenges (10). However, the long-term consequences for employees remain unclear. Moreover, there is a lack of studies investigating how the consequences for employees and service users vary depending on how CWW are organized (11). Important characteristics of compressed schedules – such as shift length, break duration between and during work periods, and the combination of extended and shorter shifts – are largely unexplored. For example, a key distinction must be drawn between CWW with and without compensatory long breaks. If long and short shifts are combined to cover changing staffing needs, this approach may reduce the recovery benefits that compressed schedules are meant to provide (11).

Evidence about the consequences of CWW with long shifts remains inconclusive. On the positive side, long shifts paired with extended recovery periods may enhance work–life balance by reducing the commuting frequency and affording workers longer, consolidated periods of personal time (12–14). A Norwegian qualitative study found that long shifts in nursing homes are associated with reduced stress, greater job satisfaction, continuity of care, and lower emotional exhaustion, although it did not assess the long-term consequences thereof (15). Additional time off during the workweek has also been linked to fewer sickness absence days, a relationship likely mediated by the increased opportunities for rest and recovery (16). However, a recent study utilizing workplace-level data did not support the notion that CWW influences sickness absence (17). In the construction sector, compressed schedules appear to alleviate fatigue and time pressure, although these effects were contingent upon individual expectations (18).

The downside of long shifts is nevertheless significant, especially in high-stress, caregiving roles. Negative outcomes include increased fatigue and reduced alertness over successive shifts, particularly by the end of a third or fourth long shift (19, 20). Shifts exceeding 12 hours, but not necessarily combined with a compensatory longer break, are associated with elevated risks of safety incidents (21). Importantly, European nurses working ≥12-hour shifts reported lower care quality, more care left undone, and higher rates of burnout compared to those on 8-hour schedules (22), with limited evidence of value propositions being realized (9). A recent systematic review on the consequences of a compressed workweek concluded that findings are mixed, depending on the outcomes, shift length, and alternative schedule (11). Overall, the results indicated increased sickness absence for workers with CWW, while simultaneously reporting higher satisfaction.

Significant gaps remain before we understand how CWW affects health and disease. As summarized by Bernstrøm and colleagues (11), the consequences of a CWW are complex and depend on *how* a CWW is organized, the *nature* of outcomes it produces, for *whom* the outcomes are relevant, *when* the effects occur, as well as *how* a CWW combines with additional workplace exposures. These challenges align with a recent discussion paper on working hours and health (23) emphasizing the need for register-based exposure assessment, large longitudinal studies, biomarker integration, and experimental designs such as cluster-randomized trials to illuminate underlying mechanisms and develop targeted interventions.

Compressed work schedules are increasingly used in municipal health and care, but we lack knowledge about the consequences. Future studies are needed to fill this knowledge gap, eg, by linking employee-level data on working hours to health registries or estimating turnover and retention and by testing CWW in randomized controlled trials.
